# Application of three-level linear mixed-effects model incorporating gene-age interactions for association analysis of longitudinal family data

**DOI:** 10.1186/1753-6561-3-s7-s89

**Published:** 2009-12-15

**Authors:** Gang Shi, Treva K Rice, Chi Charles Gu, Debeeru C Rao

**Affiliations:** 1 Division of Biostatistics, Washington University School of Medicine, 660 South Euclid Avenue, Box 8067, St. Louis, Missouri 63110, USA

## Abstract

Longitudinal studies that collect repeated measurements on the same subjects over time have long been considered as being more powerful and providing much better information on individual changes than cross-sectional data. We propose a three-level linear mixed-effects model for testing genetic main effects and gene-age interactions with longitudinal family data. The simulated Genetic Analysis Workshop 16 Problem 3 data sets were used to evaluate the method. Genome-wide association analyses were conducted based on cross-sectional data, i.e., each of the three single-visit data sets separately, and also on the longitudinal data, i.e., using data from all three visits simultaneously. Results from the analysis of coronary artery calcification phenotype showed that the longitudinal association tests were much more powerful than those based on single-visit data only. Gene-age interactions were evaluated under the same framework for detecting genetic effects that are modulated by age.

## Background

There is considerable evidence suggesting that genetic effects are modulated by age on some common complex traits. For systolic blood pressure, Rao and colleagues demonstrated age trends in familial effects [1-3]. The effect of apo-E genotype on lipid levels was shown to be age-dependent [4]. More recently, Lasky-Su et al. demonstrated the importance of gene-age interactions in replication studies of genome-wide association results [5]. They showed that the replication of a single-nucleotide polymorphism (SNP) associated with body mass index (BMI) was successful only when gene-age interaction was incorporated in the analysis. At a methodological level, longitudinal studies that collect repeated measurements on the same subjects over time have long been considered as being more powerful and providing much better information on individual changes than cross-sectional ones. Linear mixed-effects models [6] offer excellent approaches when dealing with longitudinal data. In genetic association analysis, mixed-effects models were used to account for the familial correlation among phenotypes collected from the same pedigree [7]. It was shown by simulations [8] that such regression-type association test is more powerful than the classical transmission-disequilibrium-based tests [9]. On the other hand, longitudinal family data is still less exploited. With repeated measurements on family members, phenotypes are typically correlated across both time and pedigree members. In this work, we applied a three-level hierarchical mixed-effects model in analyzing family-based longitudinal data. Association tests of genetic main effect as well as gene-age interactions were formulated under the same framework. We used the simulated phenotype data sets provided by the Genetic Analysis Workshop 16 (GAW16) Problem 3 and had the answers [10] when conducting the analyses.

## Methods

For a quantitative trait, phenotype of the *i*^th ^individual from a family measured at the *j*^th ^visit can be modeled in general as

where *β*_0 _is the mean after accounting for covariate and genetic effects, and *A*_*ij *_represents the age of the *i*^th ^individual at the *j*^th ^visit. The first three terms model the trait as a quadratic function of age at a population level, higher order terms or any other functional forms can also be applied if the phenotype so suggests. The fourth terms models the genetic main effect, where the measured genotype *g*_*i *_can be coded as dominant, additive, or recessive according to different biometric model assumptions. The fifth and sixth terms are the linear and quadratic interactions between age and genetic effects. The random effect term *a*_*ij *_accounts for familial as well as inter-visit correlations, and the last term stands for the residual, which is assumed to be independent and identically normally distributed. In longitudinal family-based studies, repeated measurements taken within a pedigree are correlated in a more complicated fashion compared with cross-sectional family studies or longitudinal studies of unrelated individuals. Repeated measurements for the same individual are temporally correlated; measurements on related individuals at each time are subject to familial correlation. More generally, measurements of related individuals at different time points are correlated as well, mostly due to the familial correlation.

Assuming independence among families, the variance-covariance matrix of marginal distribution of phenotypes is of dimension *MN × MN *for a family with *M *individuals and *N *repeated measurements. To model the variance-covariance matrix efficiently, we can exploit the structure of longitudinal family data from two distinct perspectives. One is to generalize the two-level linear mixed-effects model for cross-sectional family data [7] and treat the longitudinal family data as measurements repeated in two dimensions. The full variance-covariance matrix can be modeled as a Kronecker product of two variance-covariance matrices with dimensions *M × M *and *N × N*, respectively. The first one models the correlation across family members and the other across visits. This is mathematically equivalent to modeling the random effect *a*_*ij *_such that

where vec() stacks the columns of {*a*_*ij*_} to form a long vector, and Σ_visit _and Ω_family _represent temporal and familial variance-covariance matrices, respectively.

Alternatively, we could view the repeated measurements first cluster at the individual level and individuals as further cluster within families. Three-level mixed-effects models have been developed to model data with such hierarchical structures [11]. In this case, random effect *a*_*ij *_could be modeled as the sum of two random effect terms

where *b *represents familial effect and c_*j *_models the visit effect within a family. The second order moment of random effects *a*_*ij *_is then modeled as

where 1_*MN *_and 1_*M *_are matrices with 1 as elements and of dimension *MN *× *MN *and *M *× *M*, respectively.

With the simulated GAW 16 Problem 3 data sets we focused on the coronary artery calcification (CAC) phenotype for methodological evaluations. PROC MIXED in the computer program SAS was applied for likelihood-ratio tests. More details and examples of modeling individual growth within clusters using linear mixed-effects models may be found in Singer [12]. CAC phenotype was first transformed using a square root function, then adjusted by polynomial function of age separately within sex groups. The residuals were standardized to have a mean of zero and a variance of one. Skewness and kurtosis were evaluated to measure deviations from normality. Genotype data from the Affymetrix 50 k chip were used for genome-wide association tests. SNPs were filtered for quality control purposes. If their minor allele frequencies were less than 5%, *p*-values of Hardy-Weinberg equilibrium test were smaller than 10^-6^, or call rates less than 95%, SNPs were removed.

## Results

To evaluate the two linear random-effects models, we applied the first replication of the simulated phenotype data. No structure on temporal correlations were assumed for both the models with Kronecker and hierarchical three-level structures (i.e., unstructed 3 × 3 matrices were used), and a compound symmetric structure was assumed for the familial correlations. The tests of main effects were based on testing the fourth term *β*_3_*g*_*i *_in the model using likelihood ratio approach without any gene-age interaction terms. For additive model, it tests whether *β*_3 _= 0, and for genotype model it tests a factor with three levels. Akaike information criterion (AICs) were computed with subjects that have no missing genotype data. The -log(*p*-value) and AICs are presented in Table 1, which shows that the fitness measure of the two models are very close and that the three-level model has slightly lower AIC than the Kronecker model. *p*-Values of the tests with the two models are in general comparable, while hierarchical three-level model tends to yield slightly more significant results. In terms of computational complexity, the three-level model took about three seconds for each model fitting on a Pogo Linux sever with two AMD Opteron 270 2.0-GHz dual core central processing units and 4 GB memory, and the Kronecker model took about five minutes. Unless specified, all results are based on the three-level mixed-effects model in the rest of the paper.

**Table 1:** Major-gene SNP tests with the first replication of the simulated GAW16 data using linear mixed-effects models with Kronecker and hierarchical structures

		UN+CS^a^			UN⊗CS^b^		
			
Major gene	SNP	AIC	-Log P_1_^c^	-Log P_2_^d^	AIC	-Log P_1_	-Log P_2_
*τ*_1_	rs6743961	34355.67	0.03	0.12	34356.60	0.00	0.01
*τ*_2_	rs17714718	34365.19	5.42	6.15	34365.66	4.88	5.63
*τ*_3_	rs1894638	34352.03	0.23	0.04	34352.82	0.24	0.06
*τ*_4_	rs1919811	34354.41	0.02	0.11	34355.51	0.47	0.07
*τ*_5_	rs213952	34372.39	>16	0.21	34373.04	>16	0.29

^a^Mixed-effects model with three-level hierarchical structure

^b^Mixed-effects model with Kronecker structure

^c^P_1_, *p*-values of genotype tests

^d^P_2_, *p*-values of additive model tests

**Figure 1:**
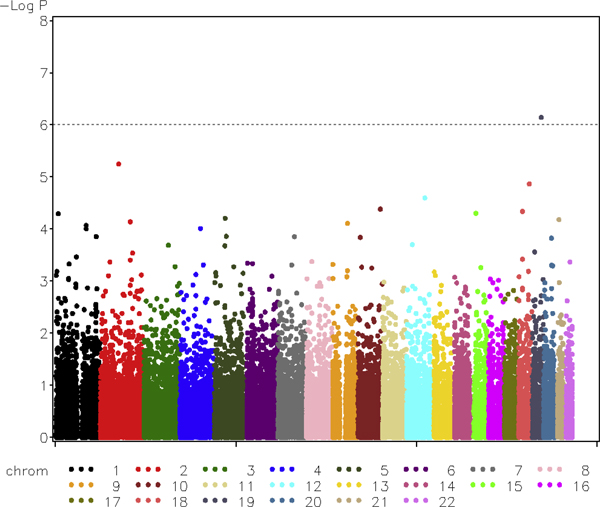
**Genome-wide association scan of CAC with the first replication of the simulated GAW16 data assuming additive model**.

Genome-wide association tests were conducted based on the first replication of CAC phenotype data set and 50 k genotype data set. Longitudinal data with three visits were applied to genotype test and test assuming additive disease model. The most significant finding from the additive model test involved SNP rs17714718 (*p*-value of 7.05 × 10^-7^), which is also the only one that passes the Bonferroni-adjusted significance level (10^-6^). This SNP is one of the five major-gene SNPs of CAC known as *τ*_2_, while other major-gene SNPs were not detected. The most significant SNP from the genotype test is rs213952 (*p*-value > 10^-16^), which is also a major gene SNP of CAC (*τ*_5_). The second significant SNP, rs17714718, also the top SNP from additive model test, has a *p*-value of 3.76 × 10^-6^, which is slightly larger than the Bonferroni adjusted significance level. A plot of the -log(*p*-values) for the additive model test is shown in Figure 1.

To compare the longitudinal analysis with cross-sectional ones, a two-level mixed-effects model [7], which modeled the familial clustering with a compound symmetric structure, was applied to analyze each single-visit data using the first replication of the simulated phenotype data. Due to the space limitation, genome-wide results were not presented. In summary, no genome-wide significant results were found in additive model tests. For the genotype test, rs213952 (*τ*_5_) passed the significance level in all three cross-sectional tests. A few SNPs appeared to be genome-wide significant in the genotype test, which are likely false-positive results. For example, SNP rs213952 has a *p*-value of 2.65 × 10^-7 ^and rs9616496 has a *p*-value of 7.23 × 10^-7 ^when tested with the second visit data set. In Table 2 we listed the -log(*p*-values) for testing rs17714718 (*τ*_2_) and rs213952 (*τ*_5_) under different scenarios. Results from the longitudinal tests are generally more significant than those from cross-sectional tests.

**Table 2:** Association tests of *τ*_2 _and *τ*_5 _with the first replication of the simulated GAW16 data using longitudinal and cross-sectional data

		3 Visits		Visit 1		Visit 2		Visit 3	
					
Major gene	SNP	-Log P_1_^a^	-Log P_2_^b^	-Log P_1_	-Log P_2_	-Log P_1_	-Log P_2_	-Log P_1_	-Log P_2_
*τ*_2_	rs17714718	5.42	6.15	1.38	1.92	3.28	3.93	1.70	2.28
*τ*_5_	rs213952	>16	0.21	6.86	0.34	6.58	0.58	11.79	1.41

^a^P_1_, *p*-values of genotype tests

^b^P_2_, *p*-values of tests of additive effects

**Figure 2:**
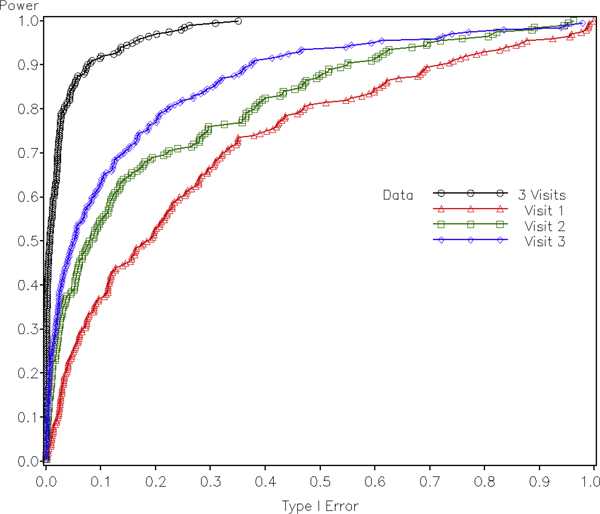
**Receiver operating characteristic of testing *τ*_2 _with 200 replicates of the simulated GAW16 data assuming additive model **.

To further compare the power of the two types of tests, we tested rs17714718 (*τ*_2_) with all 200 replications of phenotype data sets provided in the GAW16 Problem 3. Receiver operating characteristic (ROC) was plotted with empirical power as the y axis and nominal type 1 error as the x axis, where the latter was derived from thresholds and nominal distributions of the test statistics. Figure 2 illustrates the ROC curve for the tests with longitudinal and single-visit data sets assuming additive model. It can be seen that the longitudinal test with phenotypes from all the three visits is much more powerful than each cross-sectional tests. The test based on data from Visit 3 appears to be more significant than that from Visit 2; similarly, the test with Visit 2 data is more significant than that with Visit 1 data. This is exactly within expectation because CAC builds over time and the phenotype was simulated in such a way that genetic effect sizes are larger as age increases [10]. According to Table 1 in Kraja et al. [10], the average age of subjects in Visit 3 is 10 years older than that in Visit 2, which is again 10 years older than that in Visit 1. The ROC curve for the genotype test showed similar pattern, and was not included in the paper due to space limitation. We continued to test possible linear and quadratic gene-age interactions, but no significant results were found.

**Figure 3:**
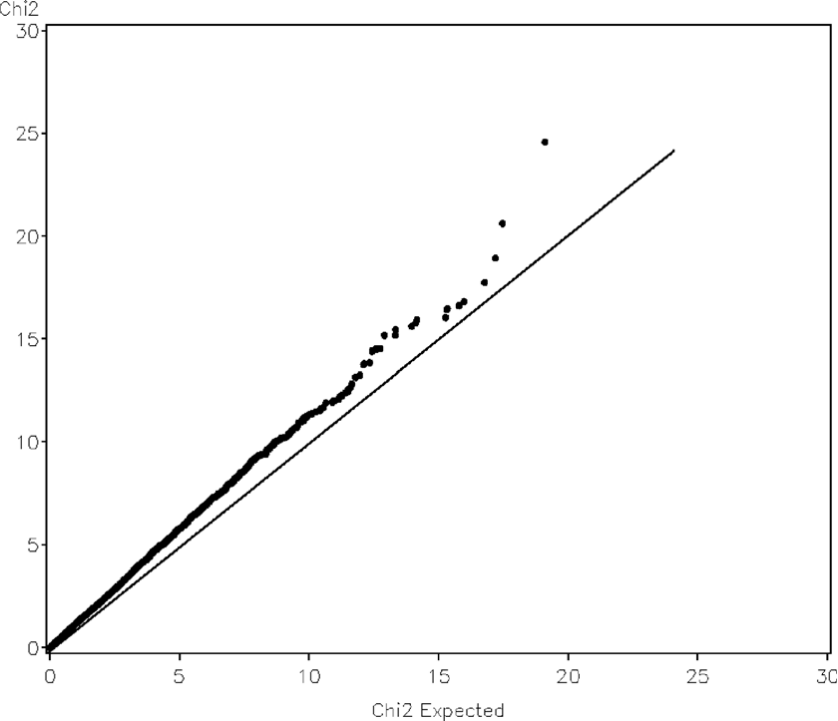
**Quantile-quantile plot of genome-wide longitudinal association scan of CAC with the first replication of the simulated GAW16 data assuming additive model**.

**Figure 4:**
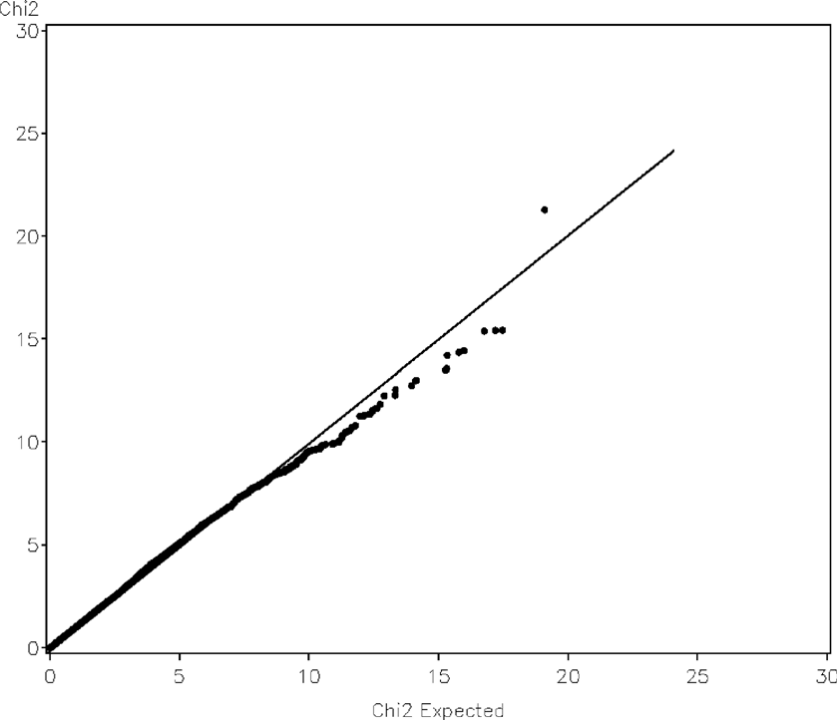
**Quantile-quantile plot of genome-wide longitudinal association scan of self-simulated null phenotype data assuming additive model**.

We examined quantile-quantile (QQ) plots of the genome-wide test statistics against their nominal distributions, which were shown in Figure 3. The QQ plot of longitudinal test with additive model was plotted versus a chi-square distribution with one degree of freedom. The distribution deviates from the expected one, and the genomic inflation factor is 1.12. For the genotype test, the inflation factor was found to be 1.08. Population substructure is a typical cause for inflated QQ plot in real data analysis. To examine null distributions of the test statistics, we simulated a set of null phenotypes. The null phenotypes within a family were simulated to be both correlated across family members and visits; compound symmetric structure was used for two types of correlation. Familial correlation was assumed to be 0.2 and inter-visit correlation for each individual was 0.6. No genetic effect was simulated in the phenotype. The QQ plot of the test for additive model with the simulated null phenotypes is shown in Figure 4. The tests statistics are now aligned with their nominal distributions quite well, and the inflation factor is 0.97. For genotype test, the inflation factor was found to be 0.98.

## Discussion

In the two linear mixed-effects models, familial correlations were assumed to have a compound symmetric structure, which is appropriate for pedigrees with first-degree relative pairs only. For pedigrees with more general relationships, such models may not be accurate and can be further generalized by introducing kinship matrix. Comparing the two linear mixed-effects models with Kronecker and hierarchical structures, the first implicitly treats each family as a single subject and phenotypes are repeatedly measured across time as well as across family members. In the three-level mixed-effects model, individuals are the subjects and measurements are repeated only across time while individuals are nested in the familial clusters.

Results from the longitudinal analysis of the three-visit phenotype data were found to be more significant than those from cross-sectional ones. Out of the five major-gene SNPs of CAC, association with rs17714718 (*τ*_2_) was detected only when using the longitudinal data (*p*-value = 7.05 × 10^-7^). SNP rs213952 (*τ*_5_) were found to be significant with both longitudinal and cross-sectional data, and the former yielded the most significant result (*p*-value > 10^-16^). None of the other major-gene SNPs were found to be significant. According to the answer to GAW 16 Problem 3 [10], *τ*_1 _was simulated to display only a minimal main effect, *τ*_2 _displays a measurable additive main effect, and *τ*_3 _and *τ*_4 _were simulated to be purely epistatic SNPs. Hence *τ*_1_, *τ*_3_, and *τ*_4 _were not significant when testing of their main effects only.

QQ plots are widely used in genome-wide association studies to measure credibility of the results. An inflated distribution of the test statistic or *p*-value may indicate possible defects in statistical analysis, e.g., population substructure, loose genotype quality control, or inappropriate statistical method. One underlying assumption of the argument is that the number of true positives is small if there will be any, hence test statistics from a genome-wide scan should represent an empirical distribution of test statistics under the null hypothesis. Inflation of the distribution should only happen in the tail due to the true-positive results, not for the whole distribution. In GAW16 Problem 3, no effect of population substructures was simulated in any phenotypes; inflated QQ plots, however, were still observed. For a complex trait like CAC, which has 17 major-gene SNPs involved directly or indirectly and 2000 polygenes spread all over the 22 chromosomes, such assumption may be worth examination. The effect sizes of all of these 2017 SNPs are so small that they may not be able to stand out at the tail of the distribution. On the other hand, the SNPs are true signals, and hence are more likely to have smaller *p*-values than other null markers. Considering further that each of these SNPs may be in linkage disequilibrium with some other SNPs, it is possible that empirical distributions may not align to their nominal distributions simply due to the complexity of the phenotype itself. In a recent genome-wide association study of dyslipidemia [13] in which population structure was adjusted using principal components, inflated QQ plots were still shown near the tail (see Figure 1 of the paper). As the title suggested, dyslipidemia is indeed a polygenic trait.

CAC is probably the most complex phenotype in the simulated GAW16 data set. All the major genes and polygenes of high-density lipoprotein, low-density lipoprotein, and triacylglyceride affect this phenotype simultaneously. Medication, diet, gene-gene, and gene-age interactions make it even more complex. In the tests of linear and quadratic gene-age interactions, no interactions were detected for any major or polygenes at a Bonferroni-corrected significance level. This may due to small marginal effects of those major gene SNPs. Also, some major gene SNPs have pure epistatic effects only, which could significantly reduce the power of test of genenotype-age interactions. In addition, because the gene-age interaction was simulated in a piece-wise linear fashion [10], this suggests that simple tests of linear or quadratic gene-age interactions may not be adequate. A function that reflects the underlying interaction form more accurately may be needed.

## Conclusion

We applied two linear mixed-effects models to analyze longitudinal family data provided by GAW16 Problem 3. The models with Kronecker and hierarchical structures yielded comparative performance in terms of goodness of fit and significance of theirs results. Longitudinal test that jointly analyze repeated phenotypes were found to be much more powerful than tests based on cross-sectional data only. Complexity of the trait itself could be a reason for inflated distribution on QQ plot, and using the correct null phenotype is suggested when evaluating distribution of test statistic under the null hypothesis.

## List of abbreviations used

AICs: Akaike information criterion; BMI: Body mass index; CAC: Coronary artery calcification; GAW: Genetic Analysis Workshop; QQ: Quantile-quantile; ROC: Receiver operating characteristic; SNP: Single-nucleotide polymorphism.

## Competing interests

The authors declare that they have no competing interests.

## Authors' contributions

GS developed the concept, carried out the analysis, interpreted the data and drafted the manuscript. TKR conducted phenotype adjustments, interpreted the data, and revised the manuscript critically. CCG acquired the data, interpreted the data and revised the manuscript critically. DCR developed the concept, interpreted the data, revised the manuscript critically, and gave final approval for publication. All authors read and approved the final manuscript.
